# Perturbation of Human T-Cell Leukemia Virus Type 1 Particle Morphology by Differential Gag Co-Packaging

**DOI:** 10.3390/v9070191

**Published:** 2017-07-19

**Authors:** José O. Maldonado, Isaac Angert, Sheng Cao, Serkan Berk, Wei Zhang, Joachim D. Mueller, Louis M. Mansky

**Affiliations:** 1 Institute for Molecular Virology, D.D.S.-Ph.D. Dual Degree Program, University of Minnesota, Minneapolis, MN 55455, USA; jmaldo@umn.edu; 2 Institute for Molecular Virology, Institute for Molecular Virology Training Program, School of Physics & Astronomy, University of Minnesota, Minneapolis, MN 55455, USA; ange0079@umn.edu; 3 Institute for Molecular Virology, Department of Diagnostic and Biological Sciences, School of Dentistry, University of Minnesota, Minneapolis, MN 55455, USA; caosheng@wh.iov.cn; 4 Institute for Molecular Virology, Department of Biomedical Engineering, University of Minnesota, Minneapolis, MN 55455, USA; sberk@umn.edu; 5 Institute for Molecular Virology, Department of Diagnostic and Biological Sciences, School of Dentistry, Characterization Facility, College of Science and Engineering, Masonic Cancer Center, University of Minnesota, Minneapolis, MN 55455, USA; zhangwei@umn.edu; 6 Institute for Molecular Virology, School of Physics & Astronomy, Masonic Cancer Center, University of Minnesota, Minneapolis, MN 55455, USA; 7 Institute for Molecular Virology, Division of Basic Sciences, School of Dentistry, Masonic Cancer Center, University of Minnesota, Minneapolis, MN 55455, USA

**Keywords:** scanning transmission electron microscopy (STEM), cryogenic transmission electron microscopy (cryo-TEM), fluorescence fluctuation spectroscopy (FFS), Gag stoichiometry, human T-cell leukemia virus type 1 (HTLV-1)

## Abstract

Human T-cell leukemia virus type 1 (HTLV-1) is an important cancer-causing human retrovirus that has infected approximately 15 million individuals worldwide. Many aspects of HTLV-1 replication, including virus particle structure and assembly, are poorly understood. Group-specific antigen (Gag) proteins labeled at the carboxy terminus with a fluorophore protein have been used extensively as a surrogate for fluorescence studies of retroviral assembly. How these tags affect Gag stoichiometry and particle morphology has not been reported in detail. In this study, we used an HTLV-1 Gag expression construct with the yellow fluorescence protein (YFP) fused to the carboxy-terminus as a surrogate for the HTLV-1 Gag-Pol to assess the effects of co-packaging of Gag and a Gag-YFP on virus-like particle (VLP) morphology and analyzed particles by cryogenic transmission electron microscopy (cryo-TEM). Scanning transmission electron microscopy (STEM) and fluorescence fluctuation spectroscopy (FFS) were also used to determine the Gag stoichiometry. We found that ratios of 3:1 (Gag:Gag-YFP) or greater resulted in a particle morphology indistinguishable from that of VLPs produced with the untagged HTLV-1 Gag, i.e., a mean diameter of ~113 nm and a mass of 220 MDa as determined by cryo-TEM and STEM, respectively. Furthermore, FFS analysis indicated that HTLV-1 Gag-YFP was incorporated into VLPs in a predictable manner at the 3:1 Gag:Gag-YFP ratio. Both STEM and FFS analyses found that the Gag copy number in VLPs produced with a 3:1 ratio of Gag:Gag-YFP was is in the range of 1500–2000 molecules per VLP. The observations made in this study indicate that biologically relevant Gag–Gag interactions occur between Gag and Gag-YFP at ratios of 3:1 or higher and create a Gag lattice structure in VLPs that is morphologically indistinguishable from that of VLPs produced with just untagged Gag. This information is useful for the quantitative analysis of Gag–Gag interactions that occur during virus particle assembly and in released immature particles.

## 1. Introduction

Human T-cell leukemia virus type 1 (HTLV-1) was the first human retrovirus identified, and it is the etiological agent of adult T-cell leukemia/lymphoma (ATLL) and the neurological disorder HTLV-1-associated myelopathy/tropical spastic paraparesis (HAM/TSP) [1,2]. It is estimated that 15 million people are infected with HTLV-1 worldwide [3,4]. Regions in the world with high prevalence levels include the Caribbean basin, South America, Central Africa, southwestern Japan, southern Africa, and the Middle East [4]. About 5% of HTLV-1 infected individuals develop an aggressive form of ATLL [5,6]. 

HTLV-1 is a deltaretrovirus and like other retroviruses, requires the expression of the Gag polyprotein—the main retroviral structural protein—to drive particle assembly and release [7]. Gag translocates from the point of translation to particle budding sites at the plasma membrane (PM) where Gag, the Gag-Pol (a Gag protein with the Pol protein fused to the carboxy-terminus of Gag), viral RNA, and the virus envelope (Env) protein assemble and result in virus particle production [8]. Aspects of this process have been suggested to involve motor proteins and the microtubule network [9], though this remains an open question. 

Lipid rafts, i.e., tightly packed saturated lipids, are typically associated with virus budding sites (reviewed by Maldonado et al. [10]), and it is at the lipid-raft associated virus budding sites that Gag is thought to form highly-ordered oligomers [11]. Gag has three structural domains: matrix (MA), capsid (CA), and nucleocapsid (NC) [12]. The N-terminal domain (NTD) of MA, which contains a hydrophobic myristic acid moiety, is responsible for the targeting and insertion of Gag into the PM [13,14]. This interaction is thought to stimulate Gag oligomerization [15] at the site of particle assembly, primarily through CA interactions [16,17] with the assistance of the viral RNA as well as cellular proteins [18,19,20,21,22]. Virus particle budding is initiated by recruitment of the endosomal sorting complexes required for transport (ESCRT) proteins [23,24].

Budding and newly released particles are typically immature and are observed via thin section transmission electron microscopy as having an electron dense Gag layer along the inner leaflet of the viral membrane. The activation of the viral protease cleaves Gag to release the mature MA, CA, and NC proteins, which results in the conversion of an immature particle to a mature infectious particle. Following virus maturation, MA remains associated with the inner leaflet of the viral membrane, NC coats the viral RNA, and this complex along with reverse transcriptase and integrase are encapsidated by the CA core [12,25]. 

We previously reported a tractable model system for HTLV-1 Gag expression and the production of HTLV-1-like particles using yellow fluorescent protein (YFP)-tagged Gag [26]. These virus-like particles (VLPs) were small, about 73 nm in diameter, and contained about 500 copies of Gag. This is in contrast to studies of authentic HTLV-1 particles and VLPs produced from expression of an untagged HTLV-1 Gag, where particles were between 110 and 113 nm in diameter, and contained 1300–1900 copies of Gag [27]. Taken together, these observations suggest that the YFP tag on the carboxy-terminus of Gag affects particle size and Gag stoichiometry. While there has been extensive use of labeled Gag proteins for studies of retroviral assembly, there is a paucity in the literature regarding their impact on Gag stoichiometry and particle morphology.

In this study, we sought to investigate whether HTLV-1-like particle morphology would be perturbed as a result of co-packaging of Gag and Gag fused to a fluorescent protein (i.e., YFP). Gag co-packaging is biologically relevant in the retrovirus assembly pathway as HTLV-1 and retrovirus particles naturally co-package Gag and Gag-Pol. To help address this, we have used cryogenic transmission electron microscopy (cryo-TEM), scanning transmission electron microscopy (STEM), and fluorescence fluctuation spectroscopy (FFS) to investigate co-packaging of Gag and Gag-YFP. We found that ratios of 3:1 (Gag:Gag-YFP) or greater resulted in a particle morphology indistinguishable from that of VLPs produced with the untagged HTLV-1 Gag, i.e., a mean diameter of ~113 nm and a mass of 83 to 120 MDa as determined by cryo-TEM and STEM, respectively. Furthermore, STEM and FFS analyses indicated that HTLV-1 Gag-YFP was incorporated into VLPs in a predictable manner at the 3:1 ratio. Both STEM and FFS analyses found that the Gag copy number in VLPs produced with a 3:1 ratio of Gag:Gag-YFP was in the range of 1500–2000 molecules per VLP. Thus, the HTLV-1 Gag copy number and particle size distribution observed with a 3:1 ratio of Gag:Gag-YFP was similar to that in authentic HTLV-1 particles [27] and within range of that observed with other retroviruses [28,29,30,31,32,33,34,35,36,37]. Taken together, the insights gained in these studies contribute new knowledge to how differential Gag co-packaging impacts virus-like particle morphology, which can have relevance to the assembly of authentic HTLV-1 particles by co-packaging of Gag and Gag-Pol.

## 2. Materials and Methods

### 2.1. Production and Purification of HTLV-1-Like Particles

A plasmid construct expressing HTLV-1 Gag with YFP fused to the carboxy-terminus (i.e., pN3 HTLV-1 Gag-YFP) [26] and a construct expressing HTLV-1 Gag only [10] have been previously described and were used to produce VLPs, as previously described [27]. Briefly, 2.2 × 10^6^ HEK 293T cells maintained in Dulbecco’s modified Eagle’s medium (DMEM, Corning, Manassas, VA, USA) supplemented with 10% Fetal Clone III (Hyclone, Logan, UT, USA) were co-transfected with 6 µg of pN3-HTLV-1 Gag-YFP or 6 µg total of pN3-HTLV-1 Gag and pN3-HTLV-1 Gag-YFP (at ratios of 1:1, 2:1, 3:1, 4:1, and 5:1) along with an HTLV-1 envelope protein (10:1 ratio of Gag to envelope plasmids) expression construct using GeneJet (SignaGen, Gaithersburg, MD, USA) following the manufacturer’s instructions. Twenty-four hours post-transfection, 2 mL of fresh media was added to cells and incubated for another 24 h. Cell culture supernatants of transfected cells were collected 48 h post-transfection, clarified by centrifugation 3000× *g* for 5 min, and filtered through a 0.2 µm filter.

VLPs were then subjected to ultracentrifugation through an 8% OptiPrep (Sigma-Aldrich, St. Louis, MO, USA) cushion at 109,000× *g* for 1.5 h using a 50.1 Ti rotor (Beckman, Brea, CA, USA) at 4 °C. The VLP pellet was resuspended in 0.5 mL of 1× sodium chloride-Tris-EDTA (STE) buffer (10 mM Tris-CL, pH 7.4, 100 mM NaCl, 1 mM ethylenediaminetetraacetic acid (EDTA)), and overlaid onto a 4 mL 10–40% OptiPrep gradient and centrifuged to equilibrium in a SW55 Ti rotor (Beckman) at 250,000× *g* for 3 h at 4 °C. The fraction containing the concentrated particles was removed from the gradient using a hypodermic needle, diluted 10-fold in 1× STE buffer and pelleted at 195,000× *g* for 1 h in a SW55 Ti rotor at 4 °C through an 8% Optiprep cushion. Following centrifugation, the VLP pellet was resuspended in ~15 µL of 1× STE overnight at 4 °C. Samples were then used for cryo-TEM and STEM analyses. For FFS measurements, 293T cells were transfected and cell culture supernatant was collected in the manner described above. Supernatant was filtered through a 0.2 µm filter and 200 µL was added to 8-well Nunc Lab-Tek Chamber Slides (Thermo Fisher Scientific, Pittsburgh, PA, USA). Slides were then sealed to prevent sample loss due to evaporation and used in FFS analyses.

### 2.2. Cryogenic Transmission Electron Microscopy Analysis of HTLV-1-Like Particles

VLP samples were prepared for cryo-TEM analysis as previously described [26]. Briefly, concentrated VLP samples were incubated on a glow-discharged c-flat holey carbon grid (Ted Pella, Redding, CA, USA), and blotted with filter paper to remove excess sample. Grids were quickly frozen in liquid ethane [38] with a FEI Vitrobot MarkIII system. A FEI TF30 field emission gun transmission electron microscope at liquid nitrogen temperature (FEI Company, Hillsboro, OR, USA) was used to analyze frozen samples. Imaging of samples was done at a nominal magnification of 59 k at low-dose (~30 electrons/Å^2^) and 1 to 5 µm underfocus conditions using a Gatan 4 k by 4 k charged coupled device (CCD) camera (Gatan Inc., Pleasanton, CA, USA). The fluorescence image and corresponding TEM picture were recorded using an FEI iCorr installed on a FEI Tecnai Spirit TEM.

### 2.3. Measurement of Virus-Like Particle Size

Two perpendicular diameter measurements were done for each VLP, as previously described [10,26]. Cryo-TEM images were analyzed using ImageJ software (National Institutes for Health, Bethesda, MD, USA). A histogram was generated for each VLP sample using GraphPad Prism 6 software (GraphPad, La Jolla, CA, USA) to determine the particle mean diameter and size distribution.

### 2.4. Fluorescence Fluctuation Spectroscopy, Experimental Setup, and Data Analysis

Virus-like particles were analyzed on an inverted microscope (AxioObserver, Zeiss, Thornwood, NY, USA) modified for two-photon FFS with an excitation wavelength of 1000 nm provided by a titanium-sapphire laser (MaiTai, Spectra Physics, Mountain View, CA, USA). FFS measurements were performed by focusing the laser inside the VLP solution and recording the fluorescence emission as the VLPs moved in and out of the observation volume via passive diffusion. Fluorescence emission was collected by a 63× water immersion objective (Zeiss, C-Apochromat, Thornwood, NY, USA, numerical aperture = 1.2), separated from excitation light by a dichroic filter, and detected by a single-photon counting module. The photon counts were recorded with a sampling frequency of 20 kHz and stored for later analysis with routines written in IDL 8.3 (Research Systems, Boulder, CO, USA).

**Figure 1:**
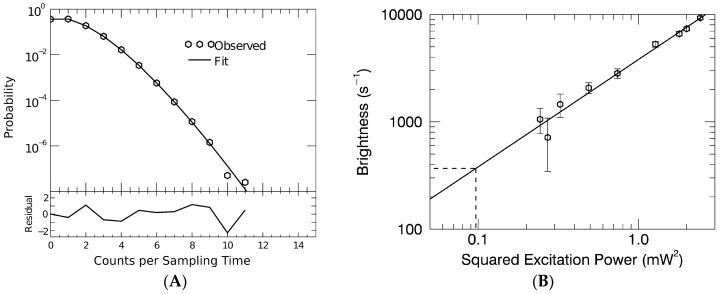
Calibration of yellow fluorescence protein (YFP) molecular brightness for measurements of Gag copy number by fluorescence fluctuation spectroscopy. (**A**) The photon counting histogram (hexagon symbols) for YFP measured at 0.47 mW excitation power is plotted together with a single species fit (solid line) with a reduced χ^2^ of 1.7; (**B**) A graph of the molecular brightness of YFP versus excitation power squared is fit to a linear relation (solid line). Individual brightness values are determined by fluorescence fluctuation spectroscopy (FFS) measurements of YFP at differing excitation powers. The reference brightness of YFP for the virus-like particle (VLP) experiment is indicated by the dashed line.

FFS measurements recorded photon counts for ~30 min and contained approximately 700 to 1300 distinct VLP events. The photon counting histogram (PCH) was calculated for each data record and fit to determine the brightness of the VLP sample as previously described [37,39]. The fit model includes background counts, two brightness species to describe VLP events, and accounts for deadtime and afterpulsing of the detector. The fit characterizes the VLPs by their effective brightness, $\lambda_{VLP}$, and particle occupation number *N* of the two brightness species. The normalized brightness *b* of the VLP sample was defined by the ratio

(1) $$b=\lambda_{VLP}/\lambda_{YFP}$$

which specifies the average number of Gag-YFP molecules per VLP. The reference brightness, *λ_YFP_*, was obtained from a separate series of FFS measurements where the excitation power was systematically varied and the brightness of a purified YFP solution was determined for each excitation power. A representative PCH of purified YFP measured at 0.47 mW excitation power and its corresponding fit to a model with a single fluorescent species is excerpted from this calibration series and shown in Figure 1A. This PCH fit recovered a brightness of 760 counts per second per molecule, an occupation number of 130 YFP, and a goodness of fit (reduced χ^2^) of 1.7. This occupation number corresponds to a concentration of ~1 μM, which is far below the reported dissociation constant of YFP dimers (~100 μM) [40], ensuring that the brightness determined by this calibration represents the brightness of true YFP monomers. The monomeric nature of the enhanced green fluorescent protein (EGFP) and related fluorescent proteins for concentrations of 10 μM or less has been experimentally verified in several FFS experiments [41,42].

The experimentally determined brightness values of the purified YFP solution were proportional to the squared excitation power as expected for two-photon microscopy (Figure 1B) [43]. Because each VLP typically contains several hundred YFP labels, VLP samples must be measured at relatively low excitation power to avoid detector saturation. However, a direct measurement of the YFP monomeric brightness is technically difficult at these low powers [37]. Therefore, we used the linear fit of the brightness versus squared power to identify the reference brightness, $\lambda_{YFP}$, at the excitation power used for VLP measurements (dashed lines, Figure 3B). This procedure identified a YFP reference brightness of $\lambda_{YFP}$ = 330/s. A second calibration method described in the literature [37], which is based on the power-dependence of the fluorescence intensity, was applied as a consistency check and confirmed the value of the YFP reference brightness (data not shown).

The diffusion time, $\tau_{D}$, of VLPs was determined from the autocorrelation function (ACF) of the photon count record and converted to the diffusion coefficient *D* by

(2) $$\tau_{D}=w_{0}^{2}/4D$$

with $w_{0}$ representing the radial beam waist of the observation volume. The average diameter *d* of VLPs was obtained from the Stokes–Einstein relation

(3) $$D=k_{B}T/3\pi \eta d$$

where η is the viscosity of water and $k_{B}T$ is the thermal energy at room temperature. Measurements were again grouped by the transfection ratio, and the median diameter of VLPs was calculated for each group. The standard deviation of the median diameter was determined by the bootstrap method.

### 2.5. Scanning Transmission Electron Microscopy Mass Measurements of Virus-Like Particles

The total mass of HTLV-1-like particles was determined (as previously shown [27]) by using a custom-built quantitative dark-field STEM developed at the Brookhaven National Laboratory (BNL, Upton, NY, USA) [44]. Purified VLPs were mixed with tobacco mosaic virus (TMV) particles, which comprise rod-like structures of known linear mass-density (13.1 kDa/Å) and are commonly used as a standard for STEM mass measurements [44,45,46]. The sample was subsequently freeze-dried onto a thin-carbon TEM grid and imaged on a low-temperature stage (−150 °C) by raster-scanning a 40-keV electron beam across the grid. The number of scattered electrons at each point within the sample was detected and recorded, yielding images with pixel intensity proportional to the mass-density at each location. The mass of individual VLPs was then determined by integrating the STEM image over each VLP and comparing against the observed mass-density of TMV particles in the same image. This comparison with TMV particles is conducted in order to ensure that mass perturbations that may be introduced during sample preparation and measurement are removed from the data collected on VLP mass [39,42,43]. For example, to account for TMV-mass changes, the raw mass values of the particles of interest are compared to the known TMV mass of 13.1 kDa/Å [30,31]. Previous analyses have shown that TMV can gain up to 20% of mass during STEM analysis during sample preparation, but can be reduced via protocol modifications [47]. Exposure to the electron beam can also cause mass loss, and this can be reduced by using the low-dose technique [44,47]. Contaminants (e.g., salts) can also contribute to a relative increase of TMV mass [48]. Following data collection, the PCMass software, developed by the BNL STEM facility, was used to analyze each virus particle’s total mass. GraphPad Prism 6 software (GraphPad, La Jolla, CA, USA) was used to generate the resulting histograms and graphs of particle mass distributions.

### 2.6. Protein and RNA Content of HTLV-1 Virus-Like Particles

The total RNA content for each sample of VLPs was determined by extracting the RNA from particle lysates with RNA columns, using Roche’s High Pure Viral RNA Kit (Roche Diagnostics, Indianapolis, IN, USA). The extracted RNA was detected by UV absorption at 260 nm using a Beckman DU-65 spectrophotometer (Beckman Coulter, Brea, CA, USA). A Thermo Scientific Pierce bicinchoninic acid (BCA) Protein Assay Kit (Thermo Fisher Scientific) was used to estimate the protein content of the same sample used to determine VLP RNA content. A bovine serum albumin protein standard and measurement of UV absorption at 562 nm were used to establish an approximate absorbance-to-concentration curve and estimate the total protein concentration of VLP samples.

## 3. Results

### 3.1. Morphology of HTLV-1-Like Particles

**Figure 2:**
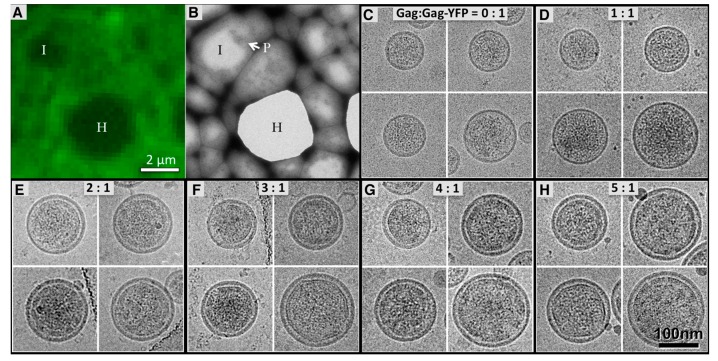
Images of Human T-cell leukemia virus type 1 (HTLV-1)-like particles as determined by fluorescence microscopy and cryogenic transmission electron microscopy (cryo-TEM). (**A**) Image recorded by using a FEI iCorr showing fluorescence signal, in green, of HTLV-1-like particles with a Gag:Gag-YFP ratio of 3:1 on a frozen transmission electron microscopy (TEM) grid. The I and H labels indicate areas of the grid that only have vitrified ice of frozen buffers, (**I**), and an empty hole on the lacey carbon film, (**H**), respectively, as observed in both panels **A** and **B**. The scale bar is also applicable to both **A** and **B**; (**B**) The TEM image of the grid at the same location of **A**, showing distribution of frozen hydrated HTLV-1-like particles (P); (**C**–**H**) Representative cryo-TEM images of HTLV-1-like particles showing size and morphology VLPs produced by transfection of HTLV-1 Gag expression plasmid(s) at 0:1 (**C**), 1:1 (**D**), 2:1 (**E**), 3:1 (**F**), 4:1 (**G**), and 5:1 (**H**) ratio of HTLV-1 Gag to HTLV-1 Gag-YFP, respectively. All HTLV-1-like particles contained an electron dense Gag lattice with gaps except for the fully labeled Gag (**C**), which is consistent with previous observation [26]. The scale bar is applicable to **C–H**.

We recently reported that HTLV-1-like particles produced by expression of HTLV-1 Gag have an electron dense ring of Gag along the inner leaflet of the viral membrane [27]. The average particle size (~110 nm) and estimated Gag copy number (1300–1600) was found to be comparable to that of authentic HTLV-1 virions. In contrast, VLPs produced from expression of Gag-YFP were smaller (~73 nm), had a lower Gag copy number (~500), and lacked an electron dense ring indicative of a properly formed Gag lattice [26]. In order to study the impact of Gag-YFP on VLP morphology, we produced VLPs by transfecting HTLV-1 Gag and Gag-YFP at various ratios, ranging from 0:1 to 5:1 of Gag:Gag-YFP (Figure 2). 

**Figure 3:**
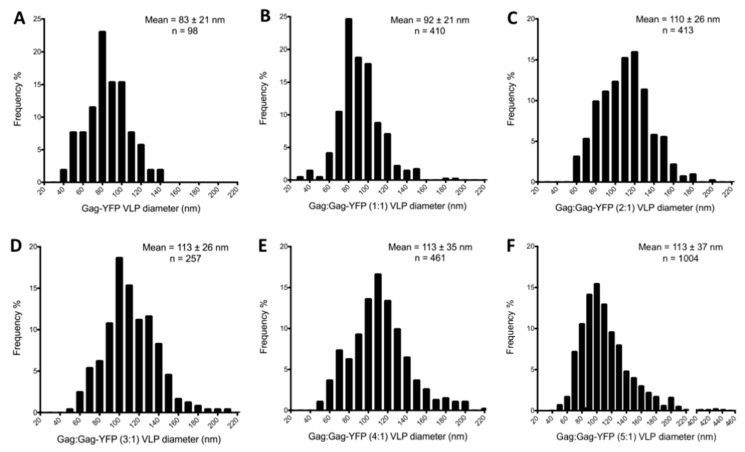
Analysis HTLV-1-like particle diameter by cryogenic transmission electron microscopy. Particle size distribution for VLPs produced by transfection of Gag expression plasmids into 293T cells at an HTLV-1 Gag to Gag-YFP ratio of 0:1 (**A**), 1:1 (**B**); 2:1 (**C**); 3:1 (**D**); 4:1 (**E**); and 5:1 (**F**).

Correlative light and electron microscopy demonstrated that the fluorescence signal co-localizes with the distribution of YFP-labeled VLPs on a frozen-hydrated TEM grid (Figure 2A,B). Figure 2C–H shows the particle morphology of VLPs produced by transfecting Gag:Gag-YFP ratios of 0:1 to 5:1. There was no Gag lattice observed in VLPs produced from Gag-YFP constructs (Figure 2C) or Gag:Gag-YFP at a ratio of 1:1 (Figure 2D). VLPs produced by transfecting HTVL-1 Gag and Gag-YFP at 2:1 ratio (Figure 2E) showed stronger electron densities inside the viral membrane but missed clear lattice configuration. At higher Gag and Gag-YFP ratios (Figure 2F–H), the VLPs were found to be spherical and contain a lattice-like arrangement indistinguishable from that observed from VLPs produced by expression of untagged HTLV-1 Gag [27]. Figure 3 shows the distribution of particle sizes produced. Interestingly, the average size of the HTLV-1-like particles increased as the ratio of Gag to Gag-YFP increased (i.e., 83 ± 21 nm to 113 ± 37 nm as illustrated in Figure 3). The particle size of Gag:Gag-YFP at ratios of 2:1 to 5:1 (i.e., 110 ± 26 nm to 113 ± 37 nm as shown in Figure 3C–F), were comparable to those reported for VLPs produced from untagged HTLV-1 Gag [27]. 

### 3.2. Gag Stoichiometry of HTLV-1-Like Particles Determined by Fluorescence Fluctuation Spectroscopy

**Figure 4:**
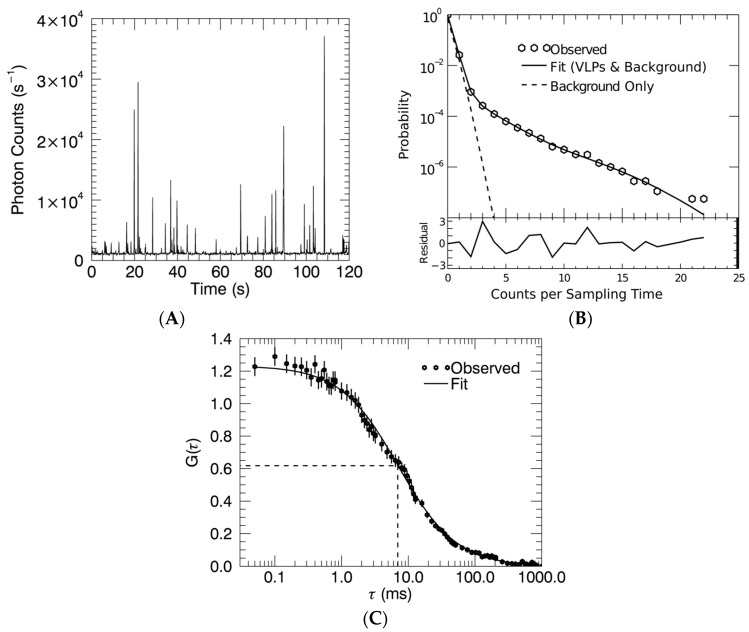
Analysis of HTLV-1-like particles by fluorescence fluctuation spectroscopy. (**A**) Shown is a two-minute excerpt from a 30-min fluorescence intensity trace. Intensities are displayed as photon counts per second; (**B**) The experimental photon counting histogram of the fluorescence intensity trace determines the probability distribution of the photon counts. Photon counting histogram analysis determines particle brightness and occupation number by a fit of this probability distribution; (**C**) This fit results in a reduced chi-square of 1.7. The experimental autocorrelation function (ACF) of the fluorescence intensity trace serves to identify the diffusion time, $\tau_{D}$, as indicated by the dashed lines.

Fluorescence Fluctuation Spectroscopy (FFS) was used to measure Gag stoichiometry and determine an average Gag copy number. The experiment records the photon counts of the fluorescence emitted as VLPs diffuse through the observation volume. A representative photon count trace of HTLV-1 Gag-YFP VLPs is shown in Figure 4A; each intensity spike corresponds to the passage of a single VLP. The photon counting histogram (Figure 4B) corresponds to the probability distribution function of all photon counts calculated from a 30-min photon count trace of HTLV-1 Gag-YFP VLPs. The VLP signal and the background signal are identified by a fit of the PCH (solid line, Figure 4B). Background counts (dashed line) dominate the left side of the histogram, while VLP events lead to high photon counts (right side of the histogram). The average brightness of the VLPs determined from the PCH fit allows estimation of the average Gag-YFP stoichiometry of VLPs by comparison to the brightness of a YFP monomer. This PCH fit identified an average brightness of 120,000 counts per second per VLP. The Gag-YFP copy number of this sample was then estimated by the ratio of the VLP brightness to the YFP monomer brightness (see Materials and Methods section) as 120,000/330 = 360. Repeated measurements (*n* = 9) of HTLV-1 Gag-YFP VLP samples resulted in a copy number of 370 ± 50.

**Figure 5:**
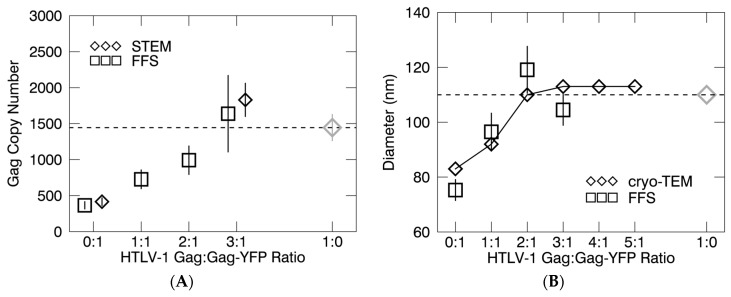
Determination of HTLV-1-like particle Gag copy number and particle diameter. (**A**) Average HTLV-1 Gag copy number and (**B**) VLP diameter as determined by fluorescence fluctuation spectroscopy (FFS) and cryogenic transmission electron microscopy (cryo-TEM) measurements.

VLPs were then harvested and analyzed from cells transfected with HTLV-1 Gag and HTLV-1 Gag-YFP at a ratio of 1:1. Copy number determinations by FFS represent only the Gag-YFP population packaged into the VLPs. Therefore, we estimated the total Gag copy number by using the assumption that the ratio of Gag to Gag-YFP within the VLPs is equivalent to the transfection ratio, resulting in a total copy number of 730 ± 130 Gag molecules per VLP. FFS measurements of VLP samples with 2:1 and 3:1 transfection ratios of Gag:Gag-YFP were then analyzed by PCH and the total Gag stoichiometry was inferred from the transfection ratio to be 990 ± 130 and 1600 ± 530 molecules of Gag per VLP, respectively. The FFS experiments identified an increase in the total Gag copy number as a function of the Gag:Gag-YFP ratio (Figure 5A), which correlated well with the increase in VLP size as determined by cryo-TEM (Figure 3). 

### 3.3. Virus-Like Particles Diameter as Determined by FFS

In addition to PCH analysis, we also investigated the autocorrelation function (ACF) of the photon counts to estimate the size of VLPs by FFS. The diffusion time, $\tau_{D}$, of a VLP sample was determined as the time when the ACF, g(τ), decayed to half its original amplitude (Figure 4C). The diffusion time was first converted to a diffusion coefficient and subsequently to a particle diameter by using the Stokes–Einstein relation (Equation (3)). This analysis was conducted on VLP samples produced from cells transfected with HTLV-1 Gag and Gag-YFP plasmid at ratios of 0:1, 1:1, 2:1, and 3:1. We determined the median diameter and its standard deviation from several measurements of VLP samples with the same ratio of labeled and unlabeled Gag. The median diameter of VLPs produced by expression in 293T cells of HTLV-1 Gag-YFP was 75 nm. Increasing the ratio of unlabeled to labeled Gag led to an increase in the particle diameter of ~110 nm for the 2:1 and 3:1 ratios of Gag:Gag-YFP as determined by cryo-TEM analysis (Figure 3 and Figure 5B). The values determined by FFS are in good agreement with the particle size determined by cryo-TEM (Figure 3 and Figure 5B).

### 3.4. Scanning Transmission Electro Microscopy Analysis of HTLV-1-Like Particle Mass

**Figure 6:**
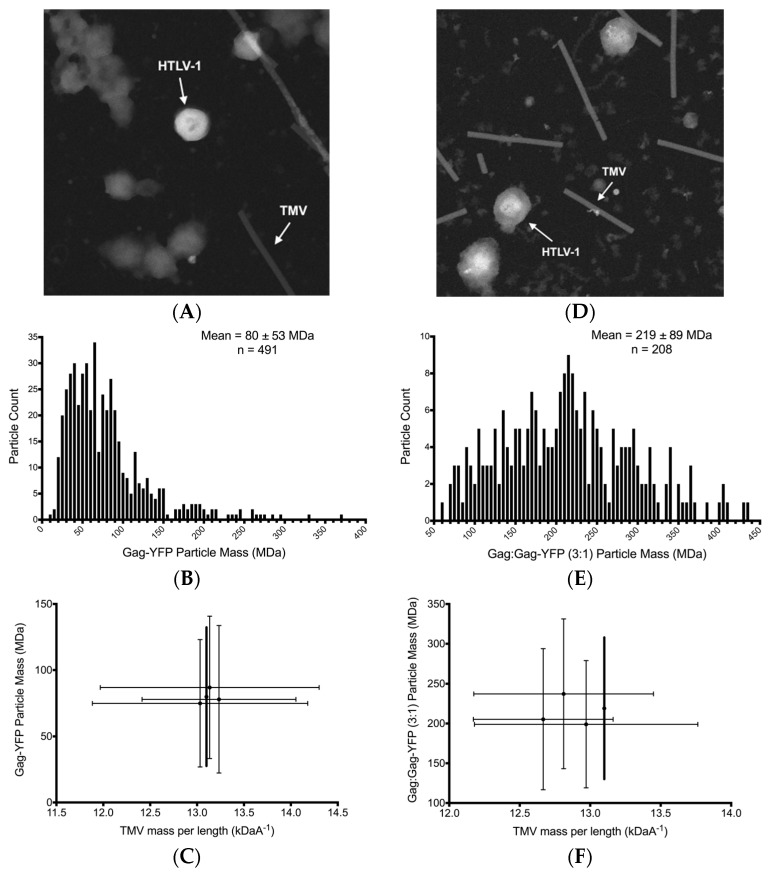
Scanning transmission electron microscopy analysis of virus-like particles produced from expression of HTLV-1 Gag-YFP and Gag:Gag-YFP (3:1). (**A**) Micrograph from scanning transmission electron microscopy of HTLV-1 Gag-YFP particles mixed with tobacco mosaic virus (TMV); (**B**) Total mass measurement distribution in MDa of HTLV-1 Gag-YFP particles; (**C**) Mass distribution per grid analyzed of HTLV-1 Gag-YFP particles; (**D**) Micrograph from scanning transmission electron microscopy of HTLV-1 Gag:Gag-YFP (3:1) particles mixed with TMV; (**E**) Total mass measurement distribution in MDa of HTLV-1 Gag:Gag-YFP (3:1) particles; (**F**) Mass distribution per grid analyzed of HTLV-1 Gag:Gag-YFP (3:1) particles. Intact HTLV-1-like particles and TMV rods are identified by arrows for both scanning transmission electron microscopy micrographs. Corrected mass of virus-like particles is indicated in bold, which was determined based upon the known TMV mass per unit length of 13.1 kDa Å^−1^. Standard deviation is indicated from three measurements.

STEM analysis was used to determine the total mass of HTLV-1-like particles as previously described [44], which allowed for the determination of the average Gag copy number per particle. Representative dark-field electron micrographs of VLPs produced by expression of HTLV-1 Gag-YFP and that of VLPs produced by co-expression of a 3:1 ratio of Gag:Gag-YFP are shown in Figure 6A,D, respectively. Only isolated and intact particles that were in the range of particle sizes observed by cryo-TEM were used for VLP mass measurements. Small contaminants are visible in the background; however, these did not affect the mass measurements based on the measured mass of TMV particles that were used as an internal control. The analyzed VLPs had a wide mass variability (Figure 6B,E), which correlates well with the VLP size range observed by using cryo-TEM (Figure 3). We determined that there was no more than a 4% increase or decrease in TMV mass (13.1 kDa Å^−1^) (Figure 6C,F). By assuming that similar mass changes exist with the HTLV-1-like particles, the TMV-corrected particle mass measurements for VLPs produced by HTLV-1 Gag-YFP (Figure 6C) and by HTLV-1 Gag:Gag-YFP at a 3:1 ratio (Figure 6F) were determined to be 80 ± 53 MDa and 219 ± 89 MDa, respectively.

### 3.5. Scanning Transmission Electron Microscopy Determination of Gag Stoichiometry in HTLV-1-Like Particles

To confirm the FFS measurements of Gag stoichiometry, we also conducted measurements using STEM. The total mass measurements of VLPs produced by expression of HTLV-1 Gag-YFP or HTLV-1 Gag:Gag-YFP particles at a ratio of 3:1 were used to estimate the average Gag copy number per VLP. The Gag:RNA mass ratio was determined to be 19.8:1 and 18.3:1 for VLPs produced with HTLV-1 Gag:Gag-YFP at a 3:1 ratio and HTLV-1 Gag-YFP, respectively, which yielded an RNA content of approximately 3% of the total VLP mass (Table 1). 

**Figure 7:**
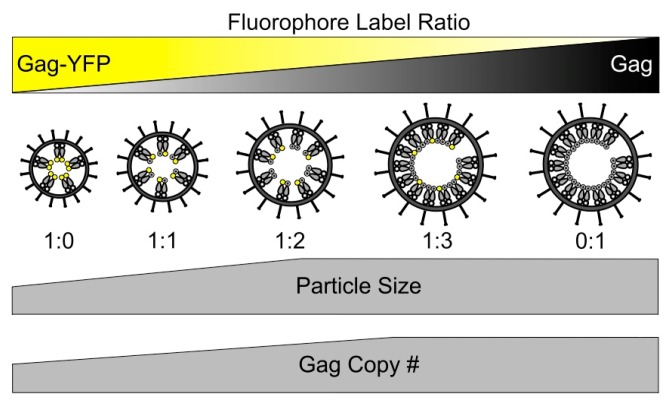
Relationship between virus-like particle size and Gag copy number to that of HTLV-1 Gag-YFP copy number. A summary of the general findings of this study are indicated with increasing ratio of HTLV-1 Gag to HTLV-1 Gag-YFP, resulting in particle diameters and Gag copy numbers that are comparable to that of particles produced with only unlabeled HTLV-1 Gag.

The average VLP diameter of 83 nm and 113 nm for VLPs produced by expression in 293T cells of HTLV-1 Gag-YFP and HTLV-1 Gag:Gag-YFP at a ratio of 3:1, respectively, and the 5 nm thickness of the viral membrane were used to estimate the number of lipid molecules in the VLP membrane. The average molecular weight of membrane lipids was estimated to be 750 Da, and it was assumed that the distance between lipid molecules is 0.85 nm [49]. Given these lipid estimates, the mass of lipids in an average size particle was estimated to be 36 MDa and 80 MDa for VLPs produced with HTLV-1 Gag-YFP and HTLV-1 Gag:Gag-YFP at a 3:1 ratio, respectively (Table 1). Based upon the assumption that the mass contribution of the Gag protein to the total VLP protein mass is similar to that of other retroviruses (i.e., in the range of 70–90%) [28,50], the mass of Gag-YFP in VLPs of average size and mass produced by expression of Gag-YFP was determined to be 29–38 MDa. Similarly, the mass of Gag and Gag-YFP in VLPs of average size and mass produced by expression of HTLV-1 Gag:Gag-YFP at a 3:1 ratio was determined to be 93–120 MDa (Table 1). VLPs of average size and mass produced by expression of Gag-YFP were calculated to contain approximately 360 to 480 Gag copies per VLP (Table 1), while VLPs of average size and mass produced by Gag:Gag-YFP at a 3:1 ratio contained approximately 1600 to 2100 copies of Gag per VLP (Table 1). A comparison of the Gag copy number as determined by STEM analysis at HTLV-1 Gag:Gag-YFP expression plasmid ratios of 0:1 and 3:1 (Table 1) indicates that the STEM and FFS measurements are in good agreement, which provides an independent confirmation of the Gag copy number determination. Taken together, elevation of the Gag ratio led to similar observations. In contrast, increasing the Gag-YFP ratio resulted in smaller VLPs, fewer Gag molecules per VLP, as well as an altered particle morphology (Figure 7).

## 4. Discussion

While HTLV-1 was the first human retrovirus discovered 35 years ago [51,52], the details of viral replication—particularly the steps involved in virus particle assembly—are poorly understood. HTLV-1 is notorious for being difficult to grow in cell culture, and the proviral sequences are prone to recombination. Given these technical limitations, knowledge of HTLV-1 replication has been lacking. We previously devised a tractable model system for the robust expression of the HTLV-1 Gag protein in mammalian cells, which resulted in efficient production of HTLV-1-like particles that have a morphology comparable to that of immature HTLV-1 particles [27]. In this study, we sought to use this model system to investigate whether HTLV-1-like particle morphology would be perturbed as a result of co-packaging of Gag and Gag fused to a fluorescent protein (i.e., YFP). Gag co-packaging is biologically relevant in the retrovirus assembly pathway as HTLV-1 and retrovirus particles naturally co-package Gag and Gag-Pol (a Gag protein with the Pol protein fused to the carboxy-terminus of Gag), reviewed in [10,53]. Thus, the insights gained in these studies have relevance to the assembly of authentic HTLV-1 particles.

HTLV-1-like particles were produced by transient transfection into 293T cells of an HTLV-1 Gag expression plasmid, an HTLV-1 Gag-YFP expression plasmid, or by co-transfection of these two plasmids at various ratios. VLP production was highly robust, due in part to the fact that the human-codon optimized versions of the Gag gene were used. We previously found that the HTLV-1 Gag-YFP construct can be used to produce VLPs [20,26]. Interestingly, in contrast to studies with HIV-1 [37], the YFP-tag was observed to affect the size and Gag stoichiometry of HTLV-1-like particles [27,54].

FFS (Figure 5A) and STEM (Table 1) measurements of VLPs produced from HTLV-1 Gag or a series of mixed transfection ratios of Gag:Gag-YFP showed that when the Gag-YFP was less than 25% of the total Gag population (3:1 ratio of Gag to Gag-YFP), the average Gag copy number was between 1600 and 2100 (Table 1), consistent with average Gag copy numbers observed in VLPs produced by transfection of 293T cells with HTLV-1 Gag alone [27]. The Gag copy number determinations from the STEM analysis relied on the assumption that regardless of the particle size, the RNA and protein content as well as the Gag:lipid ratio by mass remained constant among the VLP sample population. Given the diversity of retrovirus particle populations, these ratios are difficult to directly determine experimentally. Additionally, cryo-TEM analysis found that the VLPs produced from the co-packaging of HTLV-1 Gag:Gag-YFP at a 3:1 ratio of particles had a morphology and size that closely resembled VLPs produced by transient expression of HTLV-1 Gag in 293T cells [27]. The VLPs produced by co-packaging of Gag and Gag-YFP were found to be spherical, had a Gag lattice that follows the inner leaflet of the virus membrane (Figure 2D), and were about 113 nm in diameter (Figure 3D). Furthermore, FFS analysis independently confirmed the VLP size and Gag stoichiometry measurements made by cryo-TEM and STEM. In particular, FFS analysis indicated that HTLV-1 Gag-YFP was incorporated into VLPs in a predictable manner at the 3:1 ratio. Based upon FFS, the VLPs were found to be approximately 110 nm in diameter (Figure 5B), and contained about 1600 copies of Gag (Figure 5A).

## 5. Conclusions

The findings of this study support the conclusion that biologically relevant Gag–Gag interactions occur between Gag and Gag-YFP when they are at a ratio of 3:1 (Gag:Gag-YFP). Increasing the amount of Gag led to similar observations, while increasing the amount of Gag-YFP resulted in small VLPs, fewer Gag molecules per VLP, and an altered particle morphology (Figure 7). 

These findings are important for the analysis of Gag-YFP in living cells, particularly regarding the study of the processes involved in Gag oligomerization and Gag lattice formation. Furthermore, these studies are important for understanding the natural interactions and co-packaging between Gag and Gag-Pol, which is critical to ensure that the viral enzymes are packaged into virus particles. Proper formation of the immature Gag lattice is likely critical in order to ensure efficient Gag processing by the viral protease and formation of mature, infectious virus particles. Therefore, the ratio of co-packaging Gag and Gag-Pol is critical in order to ensure proper Gag stoichiometry as well as immature particle morphology (and likely particle infectivity). These observations support previous reports of the importance of Gag to Gag-Pol ratios in retroviral assembly [55,56]. Given the extensive use of labeled Gag proteins for studies of retroviral assembly, our observations provide important insights into the impact of Gag co-packaging on Gag stoichiometry and particle morphology.

## Figures and Tables

**Table 1:** Summary of HTLV-1-like particle mass determination and calculation of Gag copy number per particle.

Measurement		HTLV-1 Like Particle	
		Gag-YFP	Gag:Gag-YFP (3:1)
Average Diameter (nm) ^a^		83	113
Average Particle Mass (MDa) ^b^		80	219
Mass of RNA, Lipid and Protein (MDa)	RNA ^c^	2.4	6.6
	Lipid ^d^	36	80
	Total protein ^e^	42	133
Mass of Gag Molecules (MDa)	Total Gag polyprotein ^f^	29–38	93–120
	Gag	-	70–90
	Gag-YFP	29–38	23–30
Gag polyprotein copy number ^g^		360–480	1600–2100

^a^ As determined by cryogenic transmission electron microscopy; ^b^ As determined by scanning transmission electron microscopy; ^c^ Mass contributed by RNA in virus-like particles was estimated experimentally as described in the Materials and Methods; ^d^ Mass contributed by lipids was estimated from average particle size and membrane thickness; ^e^ Mass of total protein was determined by subtraction of the RNA and lipid mass from the total particle mass as determined by scanning transmission electron microscopy; ^f^ Total Gag polyprotein was estimated based upon the assumption that Gag contributes ~70–90% of the total protein mass; ^g^ The Gag polyprotein copy number represents the range of Gag copy number in a particle that has both average mass and dimensions.
